# Evaluation of Periodontitis and *Fusobacterium nucleatum* Among Colorectal Cancer Patients: An Observational Cross-Sectional Study

**DOI:** 10.3390/healthcare12212189

**Published:** 2024-11-04

**Authors:** Anna Antonacci, Cinzia Bizzoca, Giuseppe Barile, Valeria Andriola, Leonardo Vincenti, Nicola Bartolomeo, Antonia Abbinante, Germano Orrù, Massimo Corsalini

**Affiliations:** 1 Department of Interdisciplinary Medicine, ‘Aldo Moro’, University of Bari, 70100 Bari, Italy; anna_antonacci@yahoo.it (A.A.); nicola.bartolomeo@uniba.it (N.B.); antonella.abbinante@gmail.com (A.A.); 2 Department of General Surgery “Ospedaliera”, Polyclinic Hospital of Bari, 70100 Bari, Italy; dr.cinzia.bizzoca@gmail.com (C.B.); valeria.andriola@gmail.com (V.A.); massimo.corsalini@uniba.it (M.C.); 3 General Surgery Unit, National Institute of Gastroenterology IRCCS Saverio de Bellis, Research Hospital, Via Turi 27, 0013 Bari, Italy; dr.leonardo.vincenti@gmail.com; 4 Department of Surgical Sciences, University of Cagliari, 09124 Cagliari, Italy; orru@unica.it

**Keywords:** colorectal cancer, periodontitis, oral dysbiosis, *F. nucleatum*, volatile sulfur compounds

## Abstract

Background: Periodontitis has been associated with an increased risk of CRC, as well as a worse prognosis due to increased inflammation mediators and carcinogenic factors. Moreover, direct and indirect virulence factors from periodontal pathogens, such as *Fusobacterium nucleatum*, could play a pivotal role in malignant transformation and progression. This cross-sectional study aims to evaluate the presence and the stage of periodontitis in a cohort of patients with CRC. The secondary aim is to assess the presence of *F. nucleatum* and its relationship with patients’ general characteristics, concomitant pathologies, tumor characteristics, and drug therapy. Materials and Methods: Patients affected by CRC underwent dental examination and periodontal charting with the “North Carolina” probe to assess the presence and stage of periodontitis, according to the new classification of periodontal diseases of the World Workshop of the European Federation of Periodontology (EFP) and the American Academy of Periodontology (AAP) 2017. *F. nucleatum* presence was assessed by a dorsal tongue swab and related to the patient’s general characteristics, concomitant pathologies, tumor characteristics, and drug therapy. Results: Periodontal disease was found in 94.3% of I/II CRC stage patients and 100% of III/IV CRC stage patients. Severe periodontitis was found in 76% of the advanced CRC stage and 87.9% of patients with initial CRC, while initial periodontitis was found in 12.1% of initial CRC and 24% of late CRC stages, respectively, without significant differences. *F. nucleatum* presence showed no correlation between the patient’s and tumor’s characteristics, comorbidities, and drug assumed. Conclusions: Periodontal disease showed a high prevalence among CRC patients. Moreover, severe periodontitis has a higher prevalence in CRC patients compared to initial periodontitis. *F. nucleatum* presence was unrelated to CRC stage, site, other comorbidities, and drug therapies. With these data, it is not possible to admit a direct relationship between CRC and periodontal disease, but further case–control studies must be carried out to further prove this aspect. Preventive and operative targeted strategies to maintain a healthy oral status are suggested in CRC patients.

## 1. Introduction

Colorectal cancer (CRC) is one of the most common cancers in both males and females, accounting for up to 10% of cancers worldwide [1]. In recent decades, many efforts have been made to understand the factors that could cause the tumor and facilitate its progression. CRC etiology involves both hereditary and environmental factors, with the majority of cases (85–95%) being sporadic cancers [2]. It means that several exogen factors, such as dietary habits, smoking, obesity, and alcohol consumption, could trigger the intestinal carcinogenic process. 

In the literature, there is increasing evidence that intestinal microbiota and oral dysbiosis play a pivotal role in CRC initiation, progression, and metastasis [2,3]. Several studies have shown the colonization by some species of oral microbiota of the intestinal tissue samples affected by CRC [4]. The oral microbiota, as determined by the Human Microbiome Project conducted by the National Institute of Health (NIH), is represented by *Firmicutes* (mainly *Streptococcus*), *Bacteroidetes* (mainly *Prevotella*), and *Proteobacteria*, with *Fusobacteria*, *Actinobacteria*, and *Haemophilus* as dominant species [5]. Alterations in their composition are related to inflammatory diseases (i.e., rheumatoid arthritis), cardiovascular diseases, and infective status (i.e., endocarditis) [6]. The direct relation between the oral and the gut microbiota has been widely described as follows: oral dysbiosis could result in an alteration in gut microbiota [7,8]. *P. gingivalis* and *Aggregatibacter actinomycetemcomitans* are the species most linked to developing digestive cancers, such as pancreatic adenocarcinoma and CRC [9,10]. Moreover, *F. nucleatum*, *oscillibacter*, *peptostreptococcus*, *Porphyromonas*, *Roseburia*, and *Ruminococcus* were found in fecal samples from patients with CRC [11,12,13]. In particular, *P. gingivalis*, *A. actinomycetemcomitans*, and *F. nucleatum* are strongly associated with periodontitis, which belongs to Socransky’s red and orange complexes [14,15]. 

Periodontal disease is a multifactorial microbially associated inflammatory disease that is related to many systemic diseases, including the CRC [16,17]. However, the real connection and the biological pathways involved are still under debate. Different authors explored the specific role of *F. nucleatum* in CRC pathogenesis and concluded the following: it expresses a surface adhesin protein (FadA), which binds to E-cadherin, triggers β-catenin signaling pathway, and consequently stimulates the production of inflammatory cytokines and chemokines [4,14]. This mechanism could enhance CRC neoplastic cell proliferation in both in vitro and in vivo models [11,18]. Despite the well-known pathogenetic role of *F. nucleatum*, there are probably more species responsible for CRC progression. The association of several periodontal pathogen species could increase CRC incidence and worsen the treatment outcomes [3,19]. Thus, it should be desirable to develop new strategies based on the modification of the mouth pathogens and saprophytes, as well as the reduction in or elimination of oral dysbiosis, in order to improve the outcomes of medical and surgical therapies [19].

Considering this background, the main aim of this cross-sectional study is to assess the prevalence and the stage of periodontal disease among a CRC patient cohort. The secondary aim is to assess the *F. nucleatum’s* presence and relationship with patients’ general characteristics, concomitant pathologies, drug therapies, and periodontal and tumor characteristics. The null hypothesis is the absence of any relationship between periodontal disease, *F. nucleatum*, and CRC. 

## 2. Materials and Methods

### 2.1. Patient Recruitment

The patients included in this study were recruited at the General Surgery Department of the hospital “Policlinico di Bari”, Italy. All of them were diagnosed with colorectal cancer (CRC). All the patients provided written consent. This study was conducted in accordance with the Declaration of Helsinki, and the protocol was approved by the Local Ethical Committee of Hospital “IRCCS Giovanni Paolo II” (Study n. 1355/CE). Inclusion criteria were diagnosis of CRC, age > 18 years who signed the informed consent, and presence of a minimum of three natural teeth. Exclusion criteria were pregnancy, use of antibiotics in a month before the study, use of orthodontic appliances, presence of immunological diseases, or use of drugs that can affect oral tissues (e.g., phenytoin, cyclosporine, nifedipine, chronic use of non-steroidal anti-inflammatory drugs). The diagnosis of colorectal cancer was obtained through colonoscopy and pathologic confirmation (biopsy). Staging assessment was completed with a thorax–abdomen–pelvis CT scan and pelvic MRI, as defined by the Union for International Cancer Control (UICC) and American Joint Committee on Cancer (AJCC) TNM staging classification [20]. Patients underwent oncological consultation and neoadjuvant chemo-radiotherapy when indicated. Preoperative general health status and surgical risk were assessed by anesthesiologists using the ASA (American Society of Anesthesiologists) scoring system. Each patient was subjected to the following clinical steps.

### 2.2. Medical Anamnesis and Collection of Medical Records

Medical anamnesis was conducted to investigate general remote pathologies, specialistic anamnesis (past dental treatments, periodontitis familiarity, and other oral conditions), the updated pharmacological therapy, and the current pathology. 

### 2.3. Dental Examination: Orthopantomography (OPG) and Periodontal Charting

The patients were subjected to an orthopantomography, a first-line radiological exam that is useful for counting teeth and assessing the presence of dental and bone pathological processes. Then, two expert clinicians with decennial experience carried out an accurate intraoral and extraoral objective exam. Periodontal charting was performed. Each tooth is examined using the periodontal probe, “North Carolina”. The charting results were collected into a personal medical record. The diagnosis and the stage of periodontitis were conducted according to the new classification of periodontal diseases of the World Workshop of the European Federation of Periodontology (EFP) and the American Academy of Periodontology (AAP) 2017 [21]. 

The staging includes four stages of the disease:

- Stage I: Initial periodontitis;
- Stage II: Moderate periodontitis;
- Stage III: Severe periodontitis with potential tooth loss;
- Stage IV: Advanced periodontitis with extensive tooth loss and potential outcome of edentulism.

The diagnostic criteria were reported in the following table, available online at the following link: https://www.perio.org/wp-content/uploads/2019/08/Staging-and-Grading-Periodontitis.pdf (accessed on 23 October 2024) (Table 1).

### 2.4. F. nucleatum Assessment

Each biofilm sample was collected from the dorsal surface of the tongue using a disposable mini cytobrush. Sampling from this site was based on evidence that the dorsal tongue is a reservoir for periodontal pathogens, including *F. nucleatum*. The swab was placed in TAE buffer at pH 8.0 containing 20% DMSO (Dimethyl Sulfoxide, Carlo Erba, Milan, Italy) and was further used for periodontal pathogens detection. 

#### 2.4.1. Positive Control Strains 

To assess the performance of the real-time PCR, we used these strains as positive controls: (I) Fusobacterium nucleatum, CCUG 32989 (Culture Collection, University of Göteborg, Sweden) and (II) Escherichia coli ATCC 7075 (American Type Culture Collection). These strains were used as a reference for the quantification of total bacterial load by RT-PCR to perform standard curve.

#### 2.4.2. DNA Extraction

Bacterial DNA was obtained using the modified CTAB method. A 400 µL sample was added to 70 µL of 10% sodium dodecyl sulfate (SDS) and 5 µL of proteinase K at a concentration of 10 mg/mL (SIGMA—Aldrich, St. Louis, MO, USA). After vigorous mixing, this mixture was incubated for 10 min at 65 °C. Subsequently, 100 μL of NaCl [5 M] and 100 μL of CTAB/NaCl (0.274 M CTAB, Hexadecyl trimethylammonium bromide, and 0.877 M NaCl, Sigma-Aldrich, St. Louis, MO, USA) were added to the tube, which was briefly vortexed and incubated at 65 °C for 10 min. A total of 750 μL of SEVAG (Chloroform: Isoamyl Alcohol 24:1, Sigma-Aldrich, St. Louis, MO, USA) was used. The mixture was vortexed for 10 s. After centrifugation for 5 min (at 12,000 rpm), 0.6 volumes of isopropanol (Sigma-Aldrich, St. Louis, MO, USA) were added to the supernatant. After 30 min at −20 °C and centrifugation for 30 min at 12,000 rpm, the pellet was air-dried for 20 min and suspended in 20 μL of distilled water for molecular biology (Gibco, Invitrogen Paisley, Scotland, UK). A 2 μL portion was used as a DNA suspension for conventional PCR and real-time PCR reactions.

#### 2.4.3. Real-Time Quantitative PCR

The real-time PCR profile was conducted using a CFX-96 apparatus (Bio-Rad Laboratories, Hercules, CA, USA) and SYBR Premix Ex Taq Kit (TaKaRa-Clontech^®^, Kusatsu, Japan), according to the manufacturer’s instructions. The 0.02 mL final volume contained 1X 1XPremix Ex Taq (2X), 1X SYBR Green (10,000X), 0.22 μM of each primer, and 1 to 10 ng of DNA extract. The qPCR thermal profiles consisted of a denaturation step at 95 °C for 30 s, followed by 40 cycles of 5 s at 95 °C, 30 s at 60 °C, and 20 s at 80 °C. A standard curve of real-time PCR was generated using different DNA extracts obtained from different suspensions of *F. nucleatum* or *Escherichia coli* (total bacteria) with a concentration range of 101–107 cells/mL. The bacterial concentration in each sample was expressed as % *F. nucleatum* genomes/mL of DNA extract on total bacteria genomes. In this context, the oligonucleotide primers for real-time PCR are represented in Table 2.

### 2.5. Statistical Analysis

Shapiro–Wilk’s statistics were used to test normality for continuous variables, and an appropriate function was applied to transform those not showing a normal distribution. All variables and the square root transformation of the percentage of *F. nucleatum* have normal distribution and are, therefore, expressed as mean and standard deviation (SD). Student’s t-test and analysis of variance were used to evaluate the differences between continuous variables. Pairwise multiple comparisons were adjusted according to the Tukey correction. Pearson’s parametric coefficient was used to test the correlation between the continuous parameters of interest, and the Mardia test was used to verify multivariate normality. All tests of statistical significance were two-tailed, and *p*-values less than 0.05 were considered statistically significant. Statistical analysis was performed using the SAS/STAT^®^ Statistics version 9.4 (SAS Institute, Cary, NC, USA).

## 3. Results

The patients enrolled in this study were 67. Males represented 65.6% of the sample, and the average age of the subjects was 67.7 (SD 10.2). Table 3 shows the distribution of periodontitis between patients affected by CRC. The stage of periodontitis was divided into stages 1–2, called “initial”, and stages 3–4, named “severe”. For seven patients, the staging of CRC was not defined, and for two others, the stage of periodontitis was not determined.

To better visualize the previous results, we considered the 0, I, and II stages of CRC as “initial CRC” and III and IV stages as “advanced CRC”. The results are shown as follows (Table 4).

A high prevalence of periodontitis (94.3%) was found among the CRC patients. The percentage of initial stages of periodontitis is lower than the percentage of severe periodontitis among both initial and advanced CRC patients. Additionally, 82.8% of CRC patients showed severe periodontitis. 

Then, we determined the presence of *F. nucleatum* and its possible relation with general characteristics, concomitant pathologies, periodontal, and tumor characteristics (Table 5). 

The presence of *F. nucleatum* has no significant relationship with general characteristics, concomitant pathologies, periodontal, and CRC characteristics. The percentage of *F. nucleatum* was lower in subjects with cardiovascular disease and diabetics, but these differences are not statistically significant. Then, we decided to deepen these results after asking whether daily drug therapy has an impact on the *F. nucleatum* percentage. The results are presented in the following table (Table 6).

The percentage of *F. nucleatum* was lower in subjects taking antihypertensive or antiarrhythmics (15.0% vs. 24.4%) and in those treated with statins or other lipid-lowering drugs (16.4% vs. 21.6%). Instead, the percentage of *F. nucleatum* was higher in subjects treated for prostate hypertrophy (27.5% vs. 17.3%); in all cases, the differences were not statistically significant. 

## 4. Discussion

This observational study aimed to evaluate the prevalence of periodontitis among CRC patients, comparing the CRC stage to periodontitis presence and stage, the presence of *F. nucleatum*, relating it to multiple factors. The null hypothesis was accepted: no relationship was found between periodontitis and *F. nucleatum* with any parameter evaluated in the CRC patient’s cohort. 

To the best of our knowledge, no clinical studies evaluate the periodontal stage in an exclusively CRC patient cohort. The results obtained in the present study showed a high prevalence of CRC patients who were affected by periodontal disease: the periodontal disease was more frequent in the advanced CRC than in the initial CRC, without a significant relationship. This finding is consistent with several authors, who related the periodontal pathogens to the presence of CRC but not to their different stages [22,23,24]. Although many studies have been conducted on possible biological and genetic involved pathways, the association between CRC and periodontitis is still under debate [25], as reported by Fu et al. in their large sample study [23]. A cross-sectional study was conducted by Kim et al., who demonstrated an association between periodontitis and proximal colon neoplasms (*p* < 0.001), suggesting that periodontitis could represent a critical risk factor for developing proximal colon neoplasms [26]. This hypothesis is consistent with the results of a large sample study carried out on 77.443 women that demonstrated how tooth loss due to periodontal disease is related to a 20% risk for developing proximal CRC, by smoking or bad habits, respectively [22]. These results are supported by the case–control study of Janati et al., which reported a positive association between sporadic CRC and the presence of periodontitis [27]. 

This is the first time that periodontal severity was assessed by the new classification of periodontal diseases of the World Workshop of the European Federation of Periodontology (EFP) and the American Academy of Periodontology (AAP) 2017 [21] and compared to the CRC stages. Most of the studies considered periodontitis presence, but the cancer stage was rarely compared. The current literature already reports the correlation between periodontitis severity and the risk of developing CRC [28]. Mai et al. confirmed this through the evaluation of Oral Alveolar Crestal Bone Height (ACH) as the parameter that reflects the severity of chronic periodontitis [29]. The most similar study was carried out by Michaud et al. [30], which considers two periodontitis classifications: the first based on exclusively Clinical Attachment Levels (CALs) and the second based on the previous US Centers for Disease Control and Prevention–American Academy of Periodontology (CDC-AAP) [31]. They did not find any statistical association between the grade of periodontitis and the risk of CRC. However, considering the heterogenicity of periodontal classifications adopted and their relative results, conducting a specific comparison between these studies is very challenging. 

Besides the clinical implications, the relationship between periodontitis and CRC has been elucidated further in recent years, exploring the possible biological and molecular pathways involved. Firstly, a genetic linkage between periodontitis and CRC was found: they share a higher production of the same leader genes that regulate the cell cycle and the immune-inflammatory response [32]. An overexpression of CTNNB1, FOS, JUN, GRB2, PIK3CA, and PIK3R1 may affect the cell cycle, resulting in excessive cell proliferation, and malignant transformation, besides being related to periodontal disease development and progression [33,34]. Moreover, a bi-directional relationship is suggested by several authors because of the increased production of pro-inflammatory cytokines such as IL1B, IL4, IL6, IL10, CBL, and RELA, which are often present in the periodontitis development and CRC carcinogenesis [35,36]. 

Besides these well-ascertained molecular pathways involved, special focus must be placed on the relationship between the oral and gut microbiome, which can severely affect the etiopathogenesis of oral and bowel diseases due to their direct bacterial connection [37]. Many bacteria are shared between the gut and oral flora, both in healthy subjects and in patients affected by periodontitis, because oral bacteria can be used in multiple ways to access the gut microbiome [38]. They can use the dendritic cells and macrophages for their widespread diffusion, and on the other hand, they can directly pass through the digestive tract due to their resistance to gastric acid (as the *P. gengivalis*) and a possible impairment of gut microflora [39]. Flemer et al. analyzed the microbiota in oral swabs, fecal samples, and colon mucosa of patients affected by CRC and healthy individuals, showing that some microbiota oral species were found in fecal samples, where potential pathogens are found like *F. nucleatum*, *Peptostreptococcus stomatitis*, and *Parvimonas micra* [12]. The colonization of the intestinal mucosa by oral pathogens was infrequent in patients with increased oral colonies of *Lachnospiraceae*, such as *Anaerostipes*, *Blautia*, and *Roseburia*, demonstrating a protective role of such species in preventing CRC [2]. Dietary habits and other factors, such as smoking and antibiotic administration, could influence the prevalence of “protective” or “tumorigenic” species [40]. 

The most periodontal bacteria believed to be responsible for CRC carcinogenesis are *P. gengivalis* and the *F. nucleatum*, respectively, belonging to the red and orange Socransky complexes [15,41]. In particular, the latter promotes malignant transformation and proliferation through the increase in pro-inflammatory cytokines (IL-8, IL-10, TNF-a, and NF-kB), the suppression of the immune response, and the activation of the Wnt/ß-catenin pathway [42]. Thus, we aimed to find a possible correlation between the *F. nucleatum* presence and CRC staging, other comorbidities, and individual drug therapy. There was no significant relation between the presence of *F. nucleatum* and the analyzed parameters. This result contrasts with previous studies that suggest the *F. nucleatum* is a predictable marker of CRC disease [43] and relate the *F. nucleatum* to CRC poor prognosis because it is responsible for tumor growth, distant metastasis, and bad differentiation [44]. However, even if the relation between *F. nucleatum* and CRC is widely described, several aspects need further elucidation. In fact, our results are consistent with other authors [24,45], as no relation between *F. nucleatum*, CRC stage, and mortality has been found (*p* = 0.827), concluding that a single bacterial species cannot be identified as responsible for oral dysbiosis and periodontitis, as well as inflammatory and carcinogenetic alterations [19]. An interesting clinical study assessed the *F. nucleatum* abundance in a large sample of 105 patients affected by CRC, showing no differences in CRC stage and grade, consistent with the results obtained in this study, but significant differences regarding the site of cancer, as a high abundance of *F. nucleatum* was related to rectal cancer [45]. The site-specific microbiota was also confirmed by Gao et al. [46]. In contrast, we found no association between the *F. nucleatum* percentage and the CRC site (*p* = 0.195).

Many limitations are present in this study. Firstly, the sample size was small, as it was a convenience sample of patients affected by CRC in a general surgery department. The lack of statistical comparison between CRC and periodontitis in different stages, exclusively reporting their high prevalence is a weak point of this study. As such, with the data in our possession, we cannot prove whether periodontal diseases play a role in the etiology of CRC or prove a direct relationship because it is not a case–control study and only evaluates the prevalence of periodontal disease and *F. nucleatum* in a CRC patient cohort. The lack of follow-up does not permit us to collect information about the survival rate of these patients, which would be interesting to know. Even if the topic is well developed in the recent literature, considering the high incidence of CRC, there are few similar clinical studies to compare the results with, so drawing definitive conclusions is not possible. To confirm this relationship, further investigation on a larger cohort of patients and a statistical comparison with a control arm of healthy subjects are needed. 

Furthermore, we believe that the primary early event in the etiology of colorectal cancer (CRC) could also be the dysbiosis of oral flora, likely occurring in the tongue biofilm. This condition may occur years before the clinical signs of colon neoplasia associated with *F. nucleatum* appear. During oral dysbiosis, the oral bacteria, specifically *F. nucleatum*, increase and alter their expression profiles on the pathogenic motif. In this condition, it is highly likely that the pathogenic *F. nucleatum* strain can invade the colon rectal epithelium via the oral–intestinal axis. Given the significant time interval between oral dysbiosis and cancer signs, it is often possible to detect a normal condition in the oral cavity from a microbiological perspective in CRC patients. In this context, it is crucial to implement preventive measures for young subjects who have persistent oral dysbiosis.

## 5. Conclusions

This cross-sectional study showed a high prevalence of periodontal disease among the patients affected by CRC, especially in advanced tumoral stages. The null hypothesis was accepted: *F. nucleatum* presence was unrelated to CRC stage, site, and other comorbidities. Further case–control studies are needed to establish a direct relationship between CRC and periodontal disease. However, the authors suggest preventive and operative targeted strategies to maintain a healthy oral status in CRC patients.

## Figures and Tables

**Table 1:** AAP diagnostic criteria of periodontitis.

	Periodontitis	Stage I	Stage II	Stage III	Stage IV
Severity	Interdental Clinical Attachment Level (at site of greatest loss)	1–2 mm	3–4 mm	≥5 mm	≥5 mm
	Radiographic Bone Loss	Coronal third (<15%)	Coronal third (15–33%)	Extending to the middle third of the root and beyond	Extending to middle third of the root and beyond
	Tooth loss (due to periodontitis)	No tooth loss		≤4 teeth	≥5 teeth
Complexity	Local	Max. probing depth ≤ 4 mm Mostly horizontal bone loss	Max. probing depth ≤ 5 mm Mostly horizontal bone loss	In addition to Stage II complexity, the following were ntoed: Probing depths ≥ 6 mm Vertical bone loss ≥ 3 mm Furcation involvement Class II or III Moderate ridge defects	In addition to Stage III complexity, the following were noted: Need for complex rehabilitation due to: Masticatory dysfunction Secondary occlusal trauma (tooth mobility degree ≥ 2) Severe ridge defects Bite collapse, drifting, flaring <20 remaining teeth (10 opposing pairs)
Extent and distribution	Add to stage as descriptor	For each stage, describe extent as follows: Localized (<30% of teeth involved) Generalized Molar/incisor pattern			

**Table 2:** Oligonucleotide used in this work for the real-time PCR.

	Oligo Sequence 5′------3′	Oligo Name	GenBank Accession/Gene
*F. nucleatum*	GGCCACAAGGGGACTGAGACA	OG41	AJ133496/*16 rRNA*
	TTTAGCCGTCACTTCTTCTGTTGG	OG42	
Total bacteria count	CCAGCAGCCGCGGTA	OG33	*16S rRNA*
	GACTACCRGGGTATCTAATC	OG123	

**Table 3:** Distribution of periodontal disease (presence and stage) and CRC stage.

Parameter		Stage CRC				
		0–I (*n* = 13)	II (*n* = 22)	III (*n* = 20)	IV (*n* = 5)	*p*
Periodontitis presence		12 (92.3)	21 (95.5)	20 (100)	5 (100)	0.751
Periodontitis stage						
	Initial 1–2	2 (16.7)	2 (9.5)	5 (25.0)	1 (20.0)	0.539
	Severe 3–4	10 (83.3)	19 (90.5)	15 (75.0)	4 (80.0)	

**Table 4:** Relation between periodontal disease and CRC stage.

Periodontitis	Initial CRC (*n* = 35)	Advanced CRC (*n* = 25)	*p*	Overall CRC (*n* = 60)
Presence			0.506	
Yes	33 (94.3%)	25 (100%)		58 (96.7%)
No	2 (5.7%)	0 (0%)		2 (3.3%)
Stage			0.302	
Initial (Stage 1–2)	4 (12.1%)	6 (24.0%)		10 (17.2%)
Severe (Stage 3–4)	29 (87.9%)	19 (76.0%)		50 (82.8%)

**Table 5:** Percentage of *F. nucleatum* by patients’ general characteristics, concomitant pathologies, and periodontal and tumor characteristics.

Parameter		*n*	% *F. nucleatum* (Mean ± SD)	*p*
Sex				
	M	34	16.9 ± 17.4	0.214
	F	16	28.2 ± 24.2	
Smoke				
	0	18	19.3 ± 19.6	0.374
	1 pack of cigarettes per day	8	26.0 ± 19.6	
	2 pack of cigarettes per day	23	18.3 ± 21.0	
Cardiocirculatory diseases				
	Yes	31	16.6 ± 17.8	0.166
	No	19	27.0 ± 22.8	
Diabetes				
	Yes	9	10.5 ± 9.6	0.139
	No	41	22.8 ± 21.4	
Post-surgical complications				
	Yes	20	19.0 ± 20.9	0.524
	No	30	21.6 ± 20.1	
CRC site				
	Right	17	22.6 ± 23.1	0.195
	Left	10	16.7 ± 12.6	
	Rectum	20	17.5 ± 19.7	
	Transverse	3	41.7 ± 22.8	
CRC stage				
	0-I	11	21.5 ± 23.0	0.827
	II	14	16.8 ± 22.1	
	III	14	17.9 ± 13.9	
	IV	4	24.6 ± 21.7	
ASA				
	1–2	25	24.0 ± 22.3	0.225
	3–4	24	15.7 ± 16.7	
Periodontitis stage				
	1–2	8	31.2 ± 25.2	0.625
	3	19	19.9 ± 19.7	
	4	21	18.0 ± 19.3	

**Table 6:** Relationship between daily drug therapy and percentage of *F. nucleatum*.

Drug Category	*n*	% *F. nucleatum* (Mean ± SD)	*p*
Antiplatelets/anticoagulants			
yes	19	15.8 ± 14.5	0.488
no	30	22.3 ± 22.3	
Oral antidiabetics			
yes	7	10.6 ± 9.6	0.217
no	42	21.3 ± 20.6	
Antiarrhythmic antihypertensives			
yes	24	15.0 ± 17.7	0.055
no	25	24.4 ± 20.8	
Active on the Central Nervous System			
yes	3	31.4 ± 34.0	0.358
no	46	19.0 ± 18.8	
Diuretics			
yes	4	11.0 ± 16.7	0.227
no	45	20.6 ± 19.9	
Gastroprotective			
yes	16	18.9 ± 15.1	0.715
no	33	20.2 ± 21.8	
Insulin			
yes	3	7.6 ± 6.5	0.284
no	46	20.6 ± 20.1	
Prostatics			
yes	12	27.5 ± 20.9	0.061
no	37	17.3 ± 18.9	
Lipid-lowering			
yes	17	16.4 ± 22.4	0.090
no	32	21.6 ± 18.3	
Thyroid			
yes	3	14.6 ± 18.5	0.572
no	46	20.1 ± 19.9	

## Data Availability

Data are contained within the article.
